# Electrocatalytic Oxidation of Lignin on Cobalt Electrodes in Alkaline Media

**DOI:** 10.1021/acs.langmuir.6c01244

**Published:** 2026-06-16

**Authors:** André H. B. Dourado, Lucas D. Germano, Lívia C. S. Epifanio, Matheus Santos, Antonio A. S. Curvelo, Hamilton Varela

**Affiliations:** † Instituto de Química de São Carlos, 28133 Universidade de São Paulo, 13566-590 São Carlos, SP, Brazil; ‡ Instituo de Química, Universidade Estadual Paulista “Júlio de Mesquita Filho”, 14800-060 Araraquara, SP, Brazil

**Keywords:** Lignin Electrochemical Oxidation Reaction, Electrobiorefinery, Co Electrocatalyst

## Abstract

In the context of the global energy transition, the utilization of renewable feedstocks is imperative. The Lignin Electrochemical Oxidation Reaction (LEOR) represents a promising route to valorize biomass residue from industrial processes into high-value aromatics, such as vanillin, while simultaneously generating green hydrogen. While nickel is extensively studied for the LEOR, cobalt remains relatively unexplored. In this work, we optimized a potentiostatic electrodeposition process to fabricate cobalt electrodes with a high-surface-area petal-like morphology (approximately 5.8 × 10^19^ active sites per cm^2^_geom_). The Co electrocatalyst exhibited a high Faradaic efficiency for vanillin (19.5%)—surpassing bulk Ni electrodes—with high product selectivity. Remarkably, this performance was achieved at an overpotential 400 mV lower than that of nickel. However, stability challenges remain as the electrode underwent morphological degradation and surface area loss during operation.

## Graphical abstract

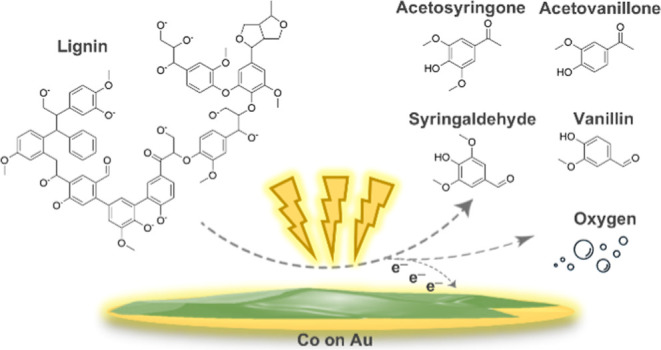

## Introduction

Lignin, along with cellulose and hemicellulose, is one of the main macromolecular components of lignocellulosic biomass.^1^ This complex macromolecule is predominantly built from three precursors—*p*-coumaryl, coniferyl, and sinapyl alcohols (Scheme 1a)—which polymerize through different linkages.^2^ The irregular and highly branched composition structure of lignin arise from the random coupling of these monomers, forming a heterogeneous web of bonds, including carbon–carbon linkages (e.g., β–β and β–5) and ether linkages (e.g., β–O–4), the latter being the most abundant in the backbone of the native lignin structure (Scheme 1b).^2,3^

**Scheme 1:**
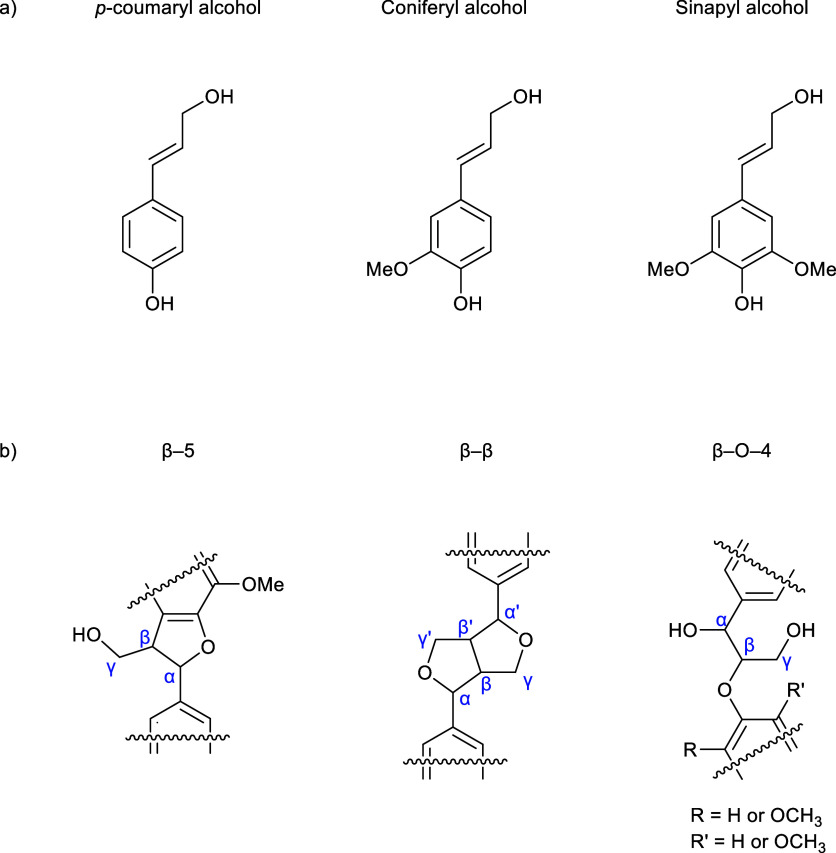
a) Representation of the Major Phenolic Monomers Found in the Lignin Kraft Structure: *p*-Coumaryl (H), Coniferyl (G), and Sinapyl Alcohols (S) and (b) Representation of the Heterogeneity of Carbon–Carbon Bonds: β–5, β–β and Ether Bonds: β–O–4

Such structural diversity and complexity give lignin a rich array of functional aromatic molecules, making it the most abundant renewable source of aromatic compounds in nature.^1,4–6^ It is worth mentioning that it is extensively released during lignocellulosic processing, particularly in pulping operations.^4,7,8^

Despite its chemical and industrial potential for obtaining aromatic compounds, such as vanillin, eugenol, and phenol, among others,^5,7,9,10^ most of the commercially available lignin is derived as a low-value byproduct from the pulp and paper industry, where it is primarily used for internal energy generation through combustion to produce steam, for thermoelectric power, and for the recovery of processing chemicals.^3^ Nevertheless, the intrinsic recalcitrance and structural heterogeneity of lignin pose significant challenges to its valorization. Unlike cellulose and hemicellulose, whose depolymerization and conversion into commodity chemicals are well-established, the selective and efficient depolymerization of lignin into value-added aromatic compounds remains a major technical bottleneck hindering its potentially broader industrial utilization.

An alternative and promising approach to overcoming such a hindrance is the electrochemical oxidation route. Indeed, the lignin electro-oxidation reaction (LEOR) can be carried out in diverse systems and a variety of electrochemical setups, in the presence of a catalyst.^11,12^ Through careful control of the oxidation parameters, steps such as overoxidation or the enhancement of the depolymerization process rate can be thoroughly controlled. In contrast to the saturated products predominantly but not exclusively observed in reductive pathways, these oxidation reactions produce more functionalized phenolics, including various carbonyl and carboxyl species.^13,14^ The former depolymerization of lignin is relatively more selective and minimizes the recondensation of lignin fragments, and the resulting lignin-derived products are generally much less oxygenated. However, due to the high population of H atoms adsorbed on the catalyst, the saturation of the carbon–carbon bonds also takes place, resulting in saturated compounds, and no aromatic products are formed.^13,15^

The LEOR in alkaline aqueous media is believed to proceed predominantly via a two-electron transfer mechanism.^16,17^ It would involve an initial electron abstraction from the phenolate anion, followed by oxidation and proton removal. Subsequent oxidation at the Cγ position would yield a Cα–Cβ bond cleavage via a retro-aldol reaction to form the target aldehyde product and carboxylic acids.^13^ Alternative oxidation at the Cα position may lead to minor byproducts, such as methyl ketones, through Cβ–Cγ bond cleavage.^12,17,18^ Nevertheless, the mechanism and the intermediates are still not fully comprehended. Since lignin depolymerization can be done by oxidative processes, the utilization of transitional metals becomes interesting due to the feasible synthetic routes, stability, and number of oxidation plurality, which, in turn, might assist in expanding this radical short lifetime, as recently demonstrated by Dourado et al.^11,19^ who utilize a metal oxide to afford and improve electrochemically the efficiency products yield, even when compared to theoretical simulations. In this specific work, the authors utilized CuO as the electrocatalyst for the LEOR, which presented significant activity and selectivity for monophenolic products generation caused by an oxygen surficial vacancies mechanism. In another recently published work by these authors,^20^ the electrocatalyst used was a metallic nickel electrode with different morphologies, applied to distinct temperature conditions toward the LEOR. By a deep comparison using *in situ* Raman spectroscopy, the authors observed that the generation of the different LEOR products was only due to different structural interfaces, which relates to the mass transport limitations of reaction intermediates. Moreover, γ-NiOOH was found to be the active species responsible for lignin electro-oxidation, and the maximum selectivity was obtained before the OER potential window. Another group suggested that NiOOH’s importance to the reaction is to activate the –OH present at the β–O–4 linkages,^21^ which would be similar to that observed for alcohol oxidation.^21,22^ Ni electrodes were also suggested to be deactivated with time,^21^ and it was suggested that OER competition should decrease the selectivity.^20,21^ The use of Faradaic efficiency (FE) is still not so common in the literature for the LEOR. However, for some cases, especially when model molecules are used, it is possible to find some calculations about it, and in the case of Ni, FE as high as 100% is presented,^23^ which is in contrast with the lower values for real lignins.^20,24^

Co metal presents an electrochemical behavior similar to that of Ni, presenting Co(0), Co­(OH)_2_, and CoOOH. This close behavior makes authors even group Co and Ni in terms of mechanism.^24^ Co offers several other advantages, such as low cost, natural abundance, simple and accessible synthesis methods, and relatively long-term chemical stability,^25–29^ and is presented as more electrochemically active than Ni.^30^ In the LEOR context, pure Co metal is not commonly found in the literature. Additionally, the features of these metal electrodes emerge as a promising material in alkaline media due to their transformation from Co­(OH)_2_ to CoOOH without drastically changing their morphological structure, withstanding the oxidative stress.^27^ The material flexibility highlights the possibility of such an electrocatalyst being incorporated into a system for the conversion of lignins.

Thus, herein, we report on the experimental study of the lignin electro-oxidation reaction on Co(0) electrodeposited films in alkaline media. The study comprises obtaining kinetic data to better understand the electrochemical behavior of lignin and evidence of adsorption of reaction intermediates on the Co films.

## Methods

### Materials

The supporting electrolyte was KOH from Sigma-Aldrich. Co­(NO_3_)_2_ was purchased from Neon Reagentes. All solutions were prepared with Milli-Q water (18.2 MΩ), and all reagents were used without further purification. The Kraft lignin samples were all extracted from a hybrid of *Eucalyptus grandis* and *Eucalyptus urophylla*, obtained from the pulp and paper industry from Suzano S.A.

### Lignin Characterization

The chemical structure of lignin was investigated by nuclear magnetic resonance (NMR) by dissolving 50 mg of the macromolecule in 0.7 mL of [D6]­DMSO. The sample was analyzed with an Agilent 500 MHz spectrometer. The measure was performed at 25 °C, over 16 scans with the gHSQCAD sequence.

The macromolecule size was also characterized by Gel Permeation Chromatography (GPC); 5 mg of Kraft lignin was solubilized in 2 mL of the eluent (sodium dihydrogen phosphate/KOH). The analysis was performed using a series of three Waters Ultrahydrogel columns (1000 Å, 250 Å e 120 Å; 7.8 mm × 300 mm) with a UV–vis detector at 254 nm and a 0.8 mL/min flow at 40 °C.

### Electrodeposition of Cobalt on Gold

The electrochemical deposition of metallic cobalt on Au electrode plates was carried out in a conventional three-electrode cell. The working electrode (WE) was composed of a 0.52 cm^2^ Au plate. The reference electrode (RE) and counter electrode (CE) were Ag/AgCl_(3 mol/L KCl)_ and a graphite rod, respectively. The solution used in the cell was 80 mL of 0.1 mol L^–1^ Co­(NO_3_)_2_. The depositions were performed under constant potential, −0.7, −0.8, or −0.9 V_Ag/AgCl_, for 1000 s, which resulted in a greenish material on Au. The greenish material was possibly a mixture of Co^0^ and its respective oxidized species (Co^2+^ and Co^3+^). Ar was purged into the electrolyte for 10 min before and during all of the electrochemical tests. All perturbation programs were controlled and recorded by an Autolab potentiostat/galvanostat model PGSTAT320N.

For the estimation of active sites, we used the Faraday constant (*F*) proportion method. Briefly, the Co on the Au electrode was positively swept at different scan rates (20 up to 1000 mV s^–1^) in 2 mol L^–1^ KOH, with Ar purged during the entire experiment, using the same electrochemical setup as the abovementioned. After the electrochemical sweeping, the oxidation peaks were integrated and divided by the respective scan rate to give the charge associated with the oxidation of Co, which is related to the reaction presented in eq 1

1 $${Co\left(OH\right)}_{2}⇌CoOOH+e^{-}+H^{+}$$

Assuming the charge is totally related to the oxidation at all the active sites and that one active site of Co is directly related with 1 electron transfer, the number of active sites was estimated by dividing the obtained charge by *F* (96,485.3321 C mol^–1^), as presented in eq 2

2 NumberofCositesmoles=(Q/96,485.3321)

Taking the average number of Co moles, which was 5.01 × 10^–5^ moles, and multiplying by the Avogadro constant (6.02214076 × 10^23^ mol^–1^), the number of active sites was determined to be 3.02 × 10^19^. Finally, this number was then normalized by the geometric area (0.52 cm^2^) of the film, giving approximately 5.8 × 10^19^ active sites per cm^2^_geom_.

### Characterization

Structural information of the electrodeposited Co on the Au surface was obtained by X-ray Diffraction (XRD) analysis. The spectra were collected by a Rigaku SmartLab SE operating with Cu Kα radiation (λ = 1.54186 Å) at 40 kV and 20 mA, in 2θ intervals ranging from 30° to 85°. The data obtained were then compared to the powder diffraction file patterns (PDFs) found at the Inorganic Crystal Structure Database (ICSD). The morphological structure of the catalysis was carried out by Scanning Electron Microscopy (SEM), Leo 440.

### Lignin Electro-Oxidation

Electrochemical behavior of lignins on Co electrodes was investigated by triangular and step potential perturbation. In this case, electrodeposited Co on Au was used as the WE, graphite rod was used as the CE, and the hydrogen reversible electrode (RHE), inserted into a Luggin capillary, was used as the RHE. The electrolyte was 10 g L^–1^ lignin Kraft in 2 mol L^–1^ KOH. Ar was purged into the electrolyte for 10 min before and during all the electrochemical tests. Electrochemical measurements were corrected for the Ohmic drop, *R* = 6.85 Ω, value estimated by the cyclic voltammetry.

### Products Quantification

For product analysis, the cell was used in a reduced volume, 5 mL. The electrode was polarized for 3 h at a constant potential. The applied values were 0.8 V_RHE_, before lignin electro-oxidation, 1.0 V_RHE_, at the CoOOH generation region, and 1.60 V_RHE_, at the oxygen evolution reaction (OER) region. The remaining liquor was acidified with H_2_SO_4_ and isolated by two extraction steps with ethyl acetate. The aqueous fraction was discarded, and the organic fraction was dried under low pressure and ambient temperature. At the end, an oil was obtained, which was solubilized in acetonitrile and analyzed by GC-FID to quantify the monoaromatic compounds. External standards were used for the preparation of analytical curves for target monophenols. From the concentration of monophenol in the lignin oil solution, the amount of produced phenols was obtained, and the Faradaic efficiency (FE) was calculated (eq. 3), considering that two electrons were needed to generate the carbonyl group.^11,24,31–33^ Using that, the FE was calculated using eq 3.

3 $$FE=\frac{nFN_{prod}}{Q_{\exp}}\times 100$$

where *n* is the number of electrons, which is 2, *F* is the Faraday constant, *N*_prod_ is the number of moles produced for a specific product, and *Q*_exp_ is the experimental total charge, obtained by the integration of the current transients. All measurements were repeated to ensure data reproducibility.

## Results and Discussion

### Lignin Characterization

Lignin HSQC spectra, shown in Figure 1, present three key regions: the aliphatic region (δC/δH 0–50 ppm/0.0–3.0 ppm), the oxygenated aliphatic region (δC/δH 50–90 ppm/6.0–2.0 ppm), and the aromatic region (δC/δH 5.5–8.0 ppm/90–135 ppm). The chemical correlation signals were assigned based on literature data.^34–37^

**Figure 1:**
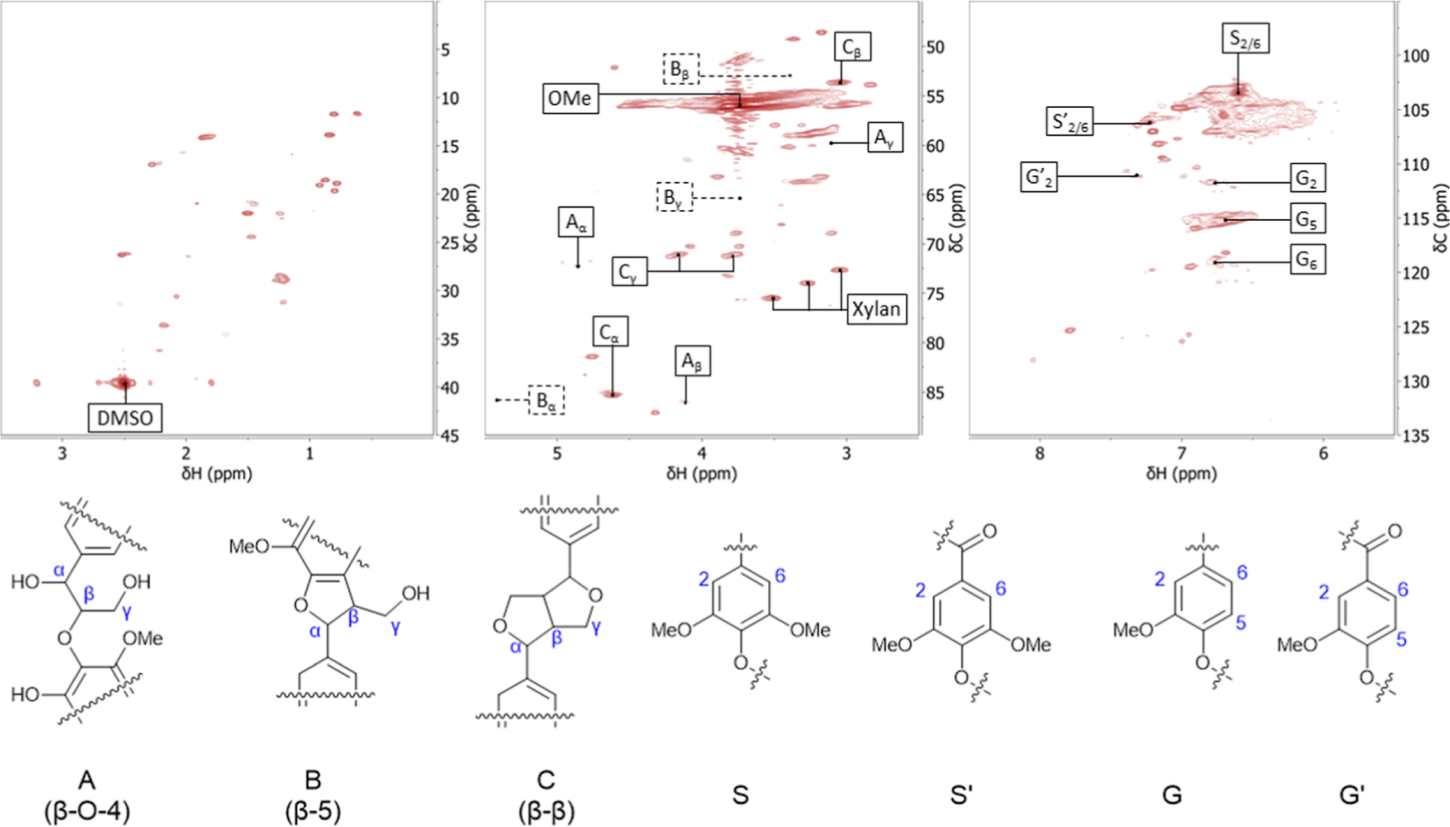
Eucalyptus Kraft lignin characterization by NMR–HSQC spectra of Kraft lignin and the identified monomeric structures and linkages.

The aliphatic region of the spectra is highly populated, indicating dehydration and possible recondensation of lignin during its isolation, as expected, since the Kraft process does not stabilize lignin fragments. In the oxygenated aliphatic region, signals corresponding to lignin interunit linkages are observed, which were quantified as the number of linkages per 100 aromatic units, which consist in the sum of the native and oxygenated aromatic moieties. From the native lignin interunit linkages, only β–O–4 and β–β were identified and quantified (4.9 and 5.2 linkages per 100 aromatic units, respectively). This result, especially the low content of β–O–4, in addition to the highly populated aliphatic region and the average Mw determined by GPC (Figure S1), indicates that native interunit linkages were cleaved and that condensation took place. Additionally, in the oxygenated aliphatic regions, signals related to the presence of polysaccharides were observed, as presented in Figure 1.

In the aromatic region, native syringyl and guaiacyl units were observed along with their C_α_-oxygenated forms. Additionally, no signals related to nonmethoxylated aromatic moieties (H) were observed. Most of the aromatic structures in Kraft lignin are S-type, with an S/G ratio of 5.9. From all S units, 9.1% are oxidized, while for G units, oxidized units represent 16.2%.

### Co Electrodeposition

The electrochemical deposition of Co on Au electrode plates was carried out by holding a potential for 1000 s. The *j*/*t* transient profiles are presented in Figure 2a.

**Figure 2:**
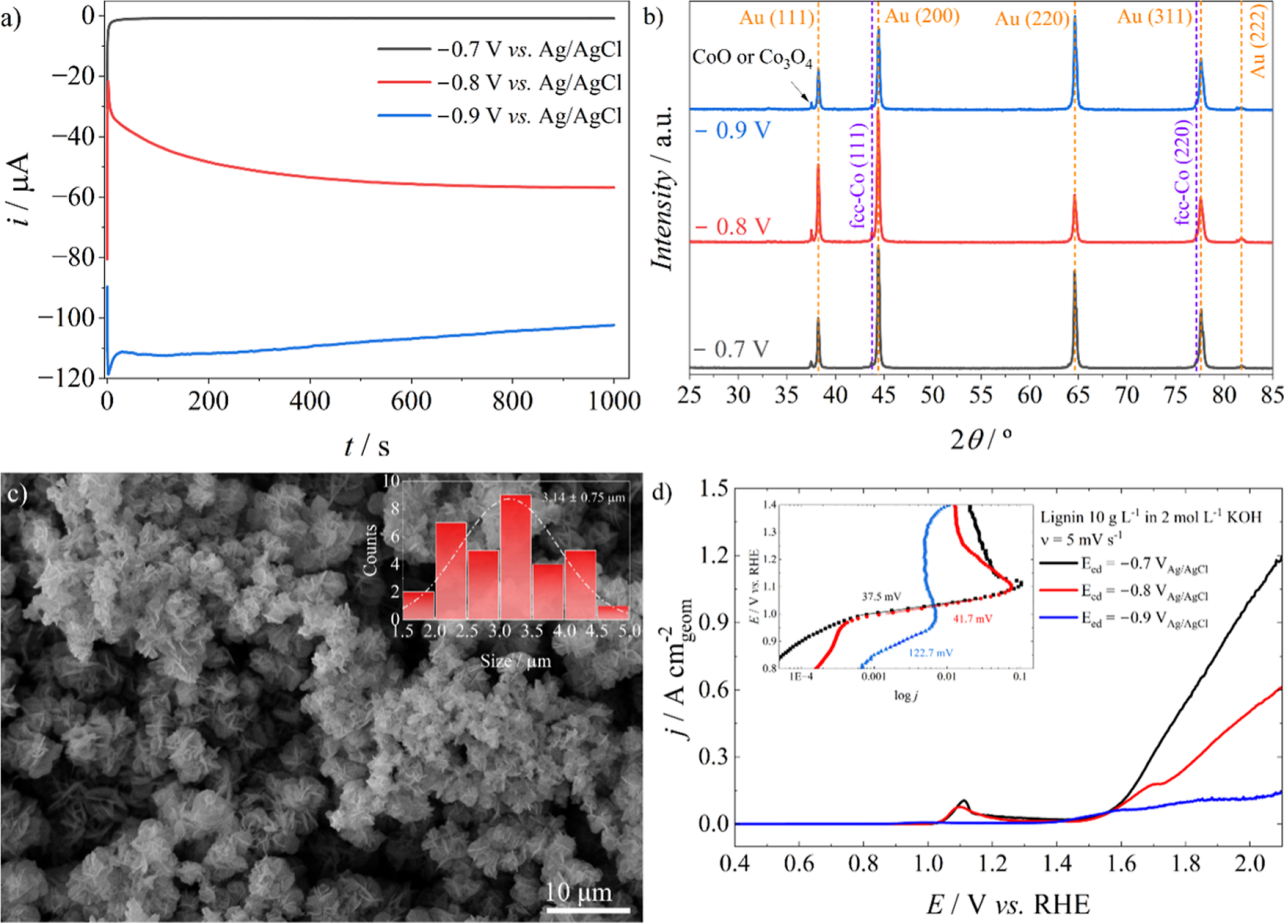
a) *j*/*t* transient recordings of Co electrodeposition on Au electrodes at an applied bias of −0.7 V (black solid line), −0.8 V (red solid line), and −0.9 V (blue solid) vs Ag/AgCl_(3 M KCl)_. (b) XRD patterns obtained for the electrodes prepared by electrodeposition at the three different potentials. The orange dashed lines represent the diffraction patterns of the gold substrate and the purple dashed lines are related to the diffraction patterns of Co films. (c) SEM micrograph of the Co electrode obtained after the electrochemical deposition at −0.8 V. Histogram of size distribution of petal-like microstructures inserted in the SEM micrograph, with an average size of 3.14 ± 0.75 μm. (d) Polarization curves of all three Co/Au electrodes in 10 g L^–1^ lignin in 2 mol L^–1^ KOH at ν = 5 mV s^–1^. The respective Tafel curves are inserted in the graph. The Tafel slopes are 37.5 mV (black dots, *E*_ed_ = −0.7 V), 41.7 mV (red dots, *E*_ed_ = −0.8 V), and 122.3 mV (blue dots, *E*_ed_ = −0.9 V). Ar was purged 10 min prior and during all the electrochemical tests.

Particularly, only at potentials −0.8 V and −0.9 V, a nucleation process associated with the current behavior was observed at the initial few seconds of the electrochemical perturbation.^38^ The shoulder-like disturbances presented in the *j*/*t* transient profiles are commonly associated with the first steps of metallic seeds agglomeration and rapid growth into nuclei, followed by a diffusion-controlled growth, which is related to the slow stabilization of the current.^39,40^

After the electrodeposition, these electrodes were characterized by XRD analysis, of which the crystallographic information of the electrodeposited Co films is shown in Figure 2b. It can be observed that the diffraction patterns of all electrodes remain very similar to each other. Four major peaks are notably observed, which are related to the Au polycrystalline substrate. The peaks at 38.24°, 44.46°, 64.64°, and 77.60° were assigned as (100), (200), (220), and (311), respectively (PDF: 01-071-3755).^41^ The remainder of the peaks at 43.74° (111) and 77.12° (220) may be attributed to the fcc-Co film (PDF: 01-090-7268);^42^ however, the isolated peak at 37.54° also suggests the presence of oxide sites (PDF: 01-076-1802),^43^ probably due to natural oxidation through samples storage and/or handling processes. It is worth noting that the sample electrodeposited at −0.8 V presented a significantly lower ratio between Au and Co signals relative to other samples, representing a more uniform Co film growth on the Au surface. The electrode prepared in the −0.8 V_Ag/AgCl_ deposition potential was also characterized by scanning electron microscopy (SEM), and the micrograph with low magnification is presented in Figure 2c. The micrograph confirms that the obtained Co film on the electrode surface presented a relatively larger surface area, presenting several pores and a rough topology. Also, it is worth mentioning that the SEM micrograph shows that the Co was electrodeposited in a petal-like morphology, with an average length size of around 3 μm, as shown in the histogram inserted.

Moreover, the electrode surface area was estimated by the double-layer capacitance (Figures S2–S4). The double-layer capacitance values obtained were 9.05, 18.43, and 12.8 μF cm_geom_^–2^ for −0.70, −080, and −0.90 V_Ag/AgCl_ deposition potentials, respectively (Figure S5). In this way, not just a more uniform Co film was obtained for the −0.8 V_Ag/AgCl_ deposition potential, but also a rougher electrode was obtained. The electrode uniformity (Figure 2c) and double-layer capacitance (Figure S5) are high points for the selection of −0.8 V_Ag/AgCl_ deposition potential as the optimal electrode. However, as presented in Figure S7, this electrode was the one that presented the last intense Co­(II)/Co­(III) conversion related to the oxidation peak around 1.0 V_RHE_, which leads to the electrode having the smallest Co active sites density (eq 2) among these three conditions (Figures S6–S8).

Even so, the comparison among these three electrodes in electrolytes without lignin makes no sense for the deposition condition selection. This is why we performed linear sweep voltammetry (LSV), as presented in Figure 2d. These data are normalized by the electrode geometric area, and in these profiles, the lowest activity is related to the electrode with the highest amount of Co, the one deposited at −0.9 V_Ag/Cl_, while the other two electrodes presented the same currents. This indicates that the electrode deposited at −0.8 V_Ag/AgCl_ presented the highest Co activity, which may be related to the fact that this electrode’s high porosity allowed the largest number of Co sites to be exposed to lignin. The Tafel slopes (Figure 2d, inset) of these two last electrodes are also very close to each other, while the first one presented the highest slope, related to a less favorable kinetics.

The electrode obtained at −0.8 V_Ag/AgCl_ was selected as the optimized electrode since it presented the most uniform morphology (Figure 2c), the highest double-layer capacitance (Figure S5), and the highest mass activity (Figures S7 and 2d).

The electrochemical surface area (ECSA) should be the correct way for electrochemical current normalization. ECSA values for electrodes such as Pt and Au are easy to obtain since one can use the H adsorption and the oxide reduction standard charges, respectively.^44–46^ When this is not the case, as for the Ni and Co electrodes,^46^ with the reversible M­(OH)_2_, MOOH conversion can be used to estimate the number of active sites, but no proper standard charge is proposed in the literature. When this is the case, many suggest the use of the double-layer capacitance as a tool for obtaining ECSA based on standard capacitance values. However, these values may vary with electrolyte, potential window applied, electrode used, and other factors.^44^ For Ni, for example, these values may vary from 10 up to 100 μF cm^–2^, with reports of 1120 cm^–2^ also being present.^45^ Because of these mismatches, the subsequent data are all normalized by the measured capacitance, shown in Figures S10–S13, which should be proportional to ECSA.

### Lignin Electro-Oxidation Reaction

After establishing the electrodeposition protocol, the Co electrodes were used for the LEOR. First, a voltammetric investigation was performed. For that, the electrode was immersed in lignin-containing solution, and the potential was disturbed triangularly (Figure 3a).

**Figure 3:**
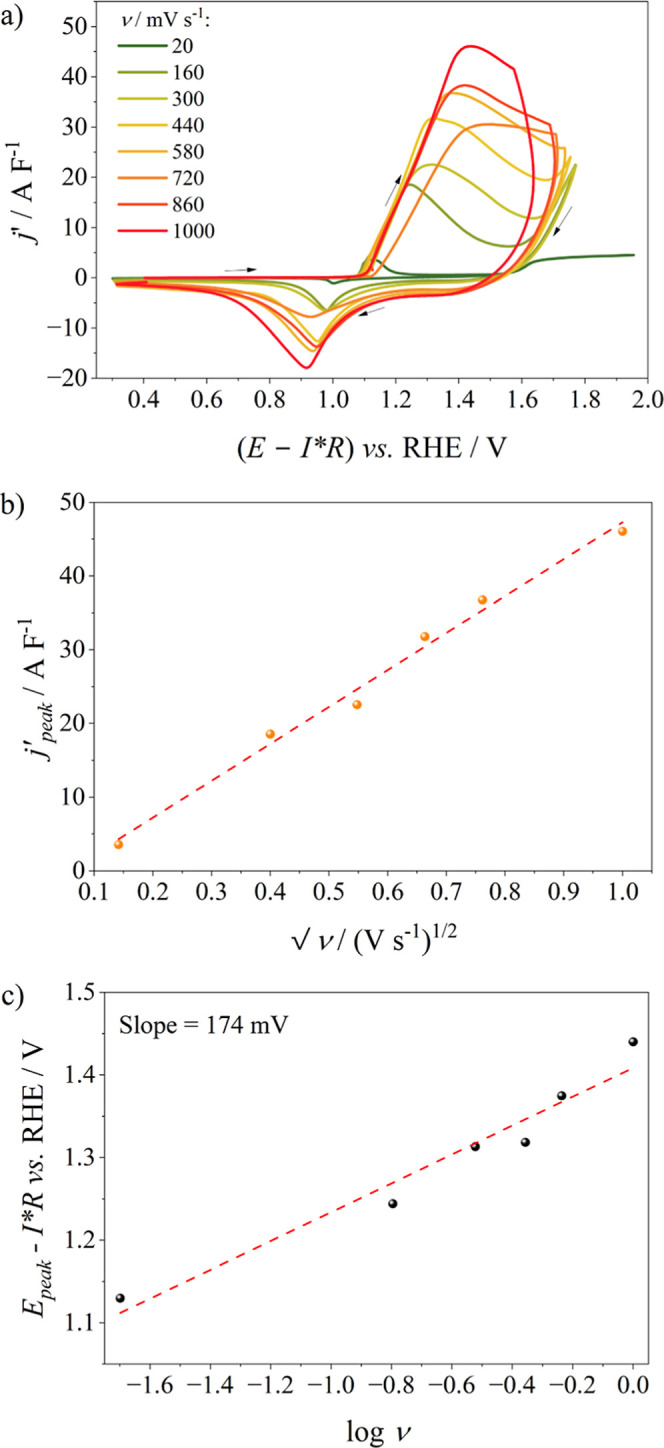
a) *j*/*E* potentiodynamic profiles at different scan rates ranging from 20 to 1000 mV s^–1^ of Co/Au in 10 g L^–1^ lignin Kraft in 2 mol L^–1^ KOH. Every profile was carried out by a single newly prepared electrode. (b) Oxidation peak currents versus the square root of the scan rates of the corresponding *j*/*E* potentiodynamic profiles. (c) Oxidation peak potentials versus log of the scan rates of the corresponding *j*/*E* potentiodynamic profiles.

Comparatively, as observed in Figure S9, the incorporation of lignin in the electrolyte resulted in a significant increase in both anodic and cathodic peak current densities. The enhancement in intensity, particularly within the oxidation peak, is directly attributed to the LEOR occurring at the Co/Au electrode surface. Notably, the oxidation peak exhibited a singular increase in current density, resulting in an overlap of the LEOR and Co oxidation processes.

Newly prepared electrodes were submitted to a triangular potential perturbation at different scan rates, resulting in the *j*/*E* potentiodynamic profiles depicted in Figure 3b. Initially, in the forward scan and at the lowest scan rate (20 mV s^–1^), the oxidation peak accounts for the sum of Co (II) oxidation to Co (III) and of lignin oxidation. In the back scan, the cathodic peak is related to the reduction of Co (III) to Co (II), and it should be free of lignin-related reactions. As such, it can be used to estimate the number of CoOOH sites available at the surface of the electrode. This estimate is easily obtained by using integrated peak charge, and considering just one electron transferred at this reaction, the number of sites can be obtained by applying the Faraday proportion, as further explained in the Methods section. As such, the average number of active sites obtained was 5.8 × 10^19^ cm^–2^.

As observed in Figure 2b, the correlation fits linearly, suggesting capacitive behavior, understood by the pseudocapacitive model, in which reaction steps that generate/consume adsorbed intermediates are related to capacitive behavior. By the same model, if this step is activation-controlled, a Tafel-like behavior of peak potential vs log ν can be obtained (Figure 3c).

The Tafel slope obtained was approximately 174 mV, which is higher than expected. If one considers a single Faradaic step, this slope should be related to the product *n*(1 – β), with *n* being the number of electrons transferred and (1 – β) being the corresponding symmetry coefficient.^47^ This value, in the present case, is much smaller than 0.5, expected for a reversible monoelectronic transfer. In the present case, *n*(1 – β) should be approximately 0.30. Electrochemical reactions are commonly described by monoelectronic steps, so in this way, considering *n* = 1 is not absurd. Since the LEOR is an overall two-electron process, this slope should be related to a reaction in which the rate-determining step is the first electron transfer.^20,47^ The slope obtained here is different from the one presented in Figure 2d. There, the value of 41.7 mV was obtained. This is an indication that some issues with mass transport could be affecting the electrochemical reaction. Because of it, we decided to apply the differential Tafel plot^48^ in the polarization curve presented in Figure 2d, and it is presented in Figure S13. For the same, the fact that the Tafel slope obtained by differentiation is not constant in the same potential window, presented in the Figure 2d inset, suggests that at that conditions, proper Butler–Volmer kinetics could not be obtained. In this way, the slope obtained in Figure 3c may be closer to the real slope.

To better comprehend this behavior, it is desirable, as well, to identify the products of this reaction. For that, we applied a fixed potential for 3 h, using just 5 mL of the electrolyte in the cell. For each experiment, a fresh electrode was prepared; the current transients are listed in Figure 4.

**Figure 4:**
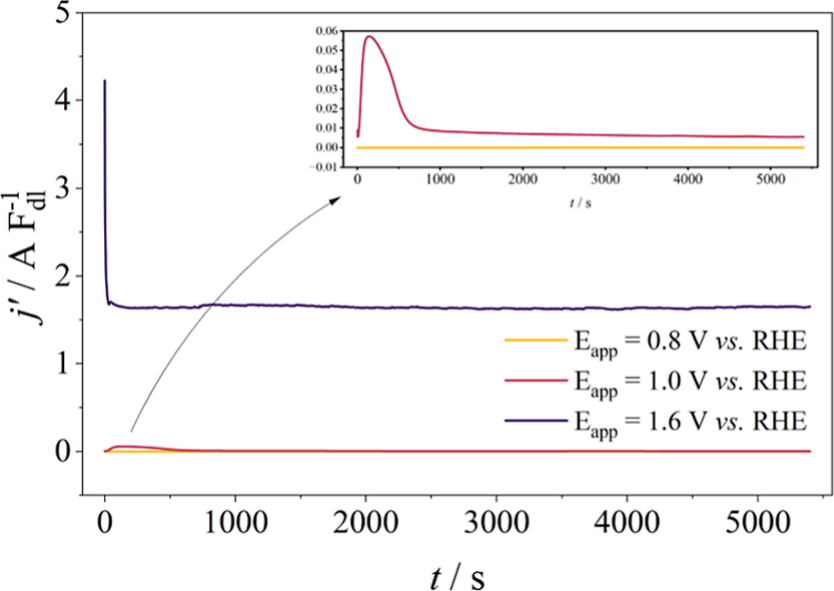
*j*/*t* transient profiles of three distinct electrodes electrodeposited in the same condition for electro-oxidation of lignin at three anodic potentials for 5400 s. The potentials applied were 0.8 V (yellow line), 1.0 V (red line), and 1.6 V vs RHE (purple line). The inset graph represents the zoom of 0.8 and 1.0 V transient curves. The *y*-axis was kept at the same scale as the main plot. Potentials presented versus RHE.

The potential values applied in Figure 4 were selected based on the *j*/*E* profile at a lower scan rate. 0.8 V_RHE_ is before the oxidation peak, while 1.0 V_RHE_ is around the peak, and 1.6 V_RHE_ is after the peak. As expected, the experiment with a potential of 0.80 V_RHE_ presented extremely low currents, so no expressive reaction was observed. At 1.0 V_RHE_, on the other hand, the transient presented an uphill direction in the few initial minutes, and then, a significant drop was observed after reaching a maximum current of approximately 0.09 mA. Thereafter, it reached a plateau for the remainder of the perturbation. Since this potential is likely to be related to the initial steps of lignin electro-oxidation peak as well as Co­(OH)_2_ oxidation to CoOOH, as observed in Figure 3a, the behavior could be associated with the diffusion of the reaction intermediate species away from the electrode’s surface, followed by the partial depletion of Co active sites all along. The biomass transport initiated with a rapid consumption of lignin, leading to the removal of Co atoms from the electrocatalyst by a coordination mechanism with reaction radical intermediates;^49^ hence, a current drop was observed. The transient for 1.6 V_RHE_ has shown an initial spike, followed by a rapid stabilization of current. Furthermore, the *j* transients may represent the cumulative electroactivity of both the oxidized Co species and the Au toward lignin oxidation. However, gold is characterized by poor electroactivity and high overpotentials for the OER and exhibits, apparently, negligible electroactivity toward the LEOR as well. Consequently, the contribution of the substrate does not significantly interfere with the Co-specific electrocatalytic performance.

The electrolyte was collected, and the products were isolated for further analysis using GC-FID. The FE (Table 1) was calculated as presented in the Methods section. The products identified were monophenolic aldehydes and ketones derived from G and S units: vanillin, acetovanillone, syringaldehyde, and acetosyringone.

**Table 1:** Faradaic Efficiencies for Monophenolic Compounds Verified by GC-FID Related to the Transients in Figure 4

Product	FE/%		
	0.8 V_RHE_	1.0 V_RHE_	1.6 V_RHE_
Vanillin	3.9	19.5	0.5
Syringaldehyde	1.19	2.3	0.4
Acetovanillone	1.5	2.4	0.2
Acetosyringone	2.4	4.5	0.2

Kraft lignin isolated from eucalyptus does not present H units, as shown in Figure 1. Thus, only products derived from G- (vanillin and acetovanillone) and S-units (syringaldehyde and acetosyringone) were identified and quantified after the LEOR.

The FE was very small for the potential before and after the oxidation peak, with the one after 1.6 V_RHE_ being the smallest. This was unexpected but can be related to the competition with other reactions, such as the oxygen evolution one. At 1.0 V_RHE_, a good total FE, 28.7% for monophenolic products, and a preference for vanillin, 19.5%, were observed. This preference for products derived from G units was not expected due to the high S/G ratio determined by HSQC. It can be related to a selective linkage breaking for the G units and/or to an instability of the S-generated products.^50^

The Co electrode has an electrochemical behavior like that of the Ni ones since both oxidize due to the presence of the equivalent oxyhydroxide. Ni has been shown by our group’s previously published work as a more selective electrode for ketones,^20^ showing an FE of around 24% for total ketones for the plane electrode and 38% for the foam one at similar temperatures used in this work; however, in the Ni work, a potential of 1.40 V_RHE_ was needed, while Co delivered around 21% FE for total aldehydes at 1.00 V_RHE_. For specifically vanillin, it was 10.2% for the planar and 16.6% for the foam electrode,^20^ while the Co one presented 19.5%.

For a more comprehensive analysis, Co/Au can be compared with other materials, as summarized by Table 2.

**Table 2:** Comparative Summary of the Metallic Electrocatalyst and Their Respective Parameters in Current Publications on the Electrochemical Depolymerization of Lignin

Electrocatalyst	Solvent	Lignin	Electrochemical parameters	Major products (yield or FE in %)
Co/Au^this work^	KOH	Kraft eucalyptus hybrid	1.0 V_RHE_	Vanillin (19.05), syringaldehyde (2.30), acetovanillone (2.40), and acetosyringone (4.5)
Ni/NiOOH^51^	NaOH	Lignosulfonate	6 mA cm^–2^	Vanillin (9.7), and acetovanillone (1.2)
Ni^52^	KOH	Organosolv	7.5 mA cm^–2^	Acetovanillone, syringaldehyde, acetylsyringone, and others (n.a.)
Ni foam^20^	NaOH	Eucalyptus	1.4 V_RHE_ and 1.8 V_RHE_ @ 285 K	Vanillin (15.62 and 28.25), acetovanillone (18.87 and 10.72), syringaldehyde (8.35 and 5.87), and acetosyringone (18.87 and 13.69)
Pb/PbO_2_ ^53^	NaOH	Cornstalk	30 mA cm^–2^	Toluene, o-xylene, anisole, and other monophenolic products (n.a.)
CuO^11^	KOH	Sugar cane bagasse Pepper lignin	Variety	Vanillin, acetovanillone, syringaldehyde, acetosyringone, *p*-hydroxybenzaldehyde, and *p*-hydroxyacetophenone (all around 25–30)
Carbon paper coated w/ Cu^54^	KOH	Kraft	1.0 V_Ag/AgCl_	Vanillin (62.4), vanillic acid (54.1), and aromatic monomers
β-PbO_2_/MWCNTs^55^	NaOH	Biorefinery	0.5 V_SHE_	Vanillin, methyl salicylate, 1,3-bis(1,1-dimethylethyl)-benzene, and 2,4-bis(1,1-dimethylethyl)-phenol (all around 3%–17%)
Ti/RuO_2_–IrO_2_ ^56^	NaOH	Kraft	2 A cm^–2^	Vanillin and vanillinc acid (n.a.)
Pb/PbO_2_ ^57^	NaOH	Bamboo	20 mA cm^–2^ @ 313.15 K	Vanillin (36.06), syringaldehyde (57.30), and *p*-coumaric acid (29.64)

For a bigger picture of the catalyst behavior, an analysis post-mortem is also of great interest. The electrodes were analyzed by SEM, XRD, and surface analysis, once again.

The SEM micrographs are presented in Figure 5a–c. As observed, the petal-like structures were clearly destroyed. The electrodes present a more planar morphology, and the surface capacitance decreased 70%, 85%, and 70% (Figures S10–S13) after the applied potentials of 0.8, 1.0, and 1.6 V_RHE_, respectively. This shows that a Co electrode is corroded by the process, because Co^3+^ can be complexed into the lignin macromolecule conformation.^49^ Probably the Co sites are being consumed during the adsorbed intermediate generation, resulting in the decrease in activity observed in Figure 4, current transient for the 1.0 V_RHE_ applied potential.

**Figure 5:**
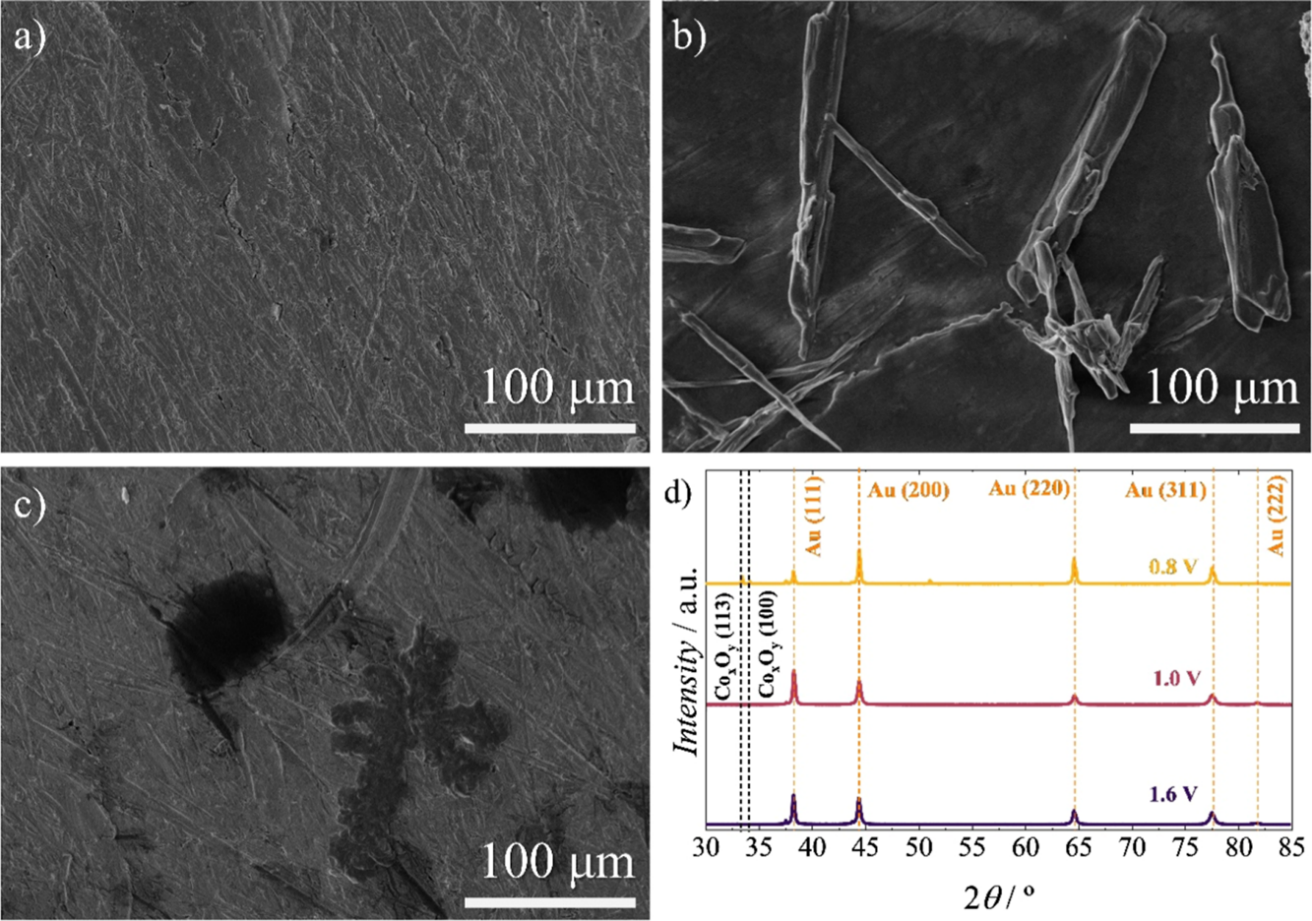
SEM micrographs of the Co electrode obtained after the electrochemical oxidation of 10 g L^–1^ lignin Kraft at (a) 0.8, (b)­1.0, and (c)­1.6 V versus RHE for 3 h. (d) XRD patterns obtained for electrodes after the lignin electro-oxidation at three different potentials: 0.8 V (yellow line), 1.0 V (red line), and 1.6 V vs Ag/AgCl_(3 M KCl)_ (purple line).

The crystallographic structure was also verified by XRD (Figure 5d).

In the initial stages of Co oxidation (near 0.8 V), there is a rapid formation of Co_*x*_O_*y*_ species related to CoOOH sites on the electrocatalyst surface, which was supported by the appearance of 2θ equal to 33.4° and 37.5° related to (113) and (100) phases, respectively (PDF: 01-076-1802, 01-071-1178 and 01-089-8399).^27,29^ Thereafter, at 1.0 V, the oxide peaks disappear, with the Au substrate peak remaining majorly. Similarly, the material diffraction pattern at 1.6 V was close to the latter. This resemblance might be related to the fact that the Co film was partially detached from the electrode’s surface. It is worth mentioning that the diffraction pattern of the Au substrate was relatively higher for the 1.6 V_RHE_ electrode when compared to the others. Additionally, the ratio between Au and Co intensities in the case of 1.6 V_RHE_ is higher than its counterparts. Both these trends correspond to proportionally more Au atoms and fewer Co ones. To address the instability of the electrodeposited films, several strategies might be used to enhance structural integrity. These approaches include substrate premodification, functionalization of the Au surface with anchoring ligands to promote Co stronger adhesion, mild temperature annealing of the films in an inert atmosphere, or synthesis of Co nanostructures for subsequent deposition onto Au substrates.

## Conclusions

It was shown that Co electrodes can be easily obtained by electrochemical deposition and that these electrodes present a good activity for the LEOR. The most interesting point observed was the selectivity of vanillin. The FE was 19.5%, at low overpotential, this being almost the only monophenolic product detected. This behavior is unlikely to be presented in the literature for most materials, showing that Co, despite not being so much investigated, presents interesting aspects. The main issue observed is the stability. The electrode is easily corroded under operando conditions, losing not just the controlled morphology but also the amount of active material over the electrode. Even so, it highlights the need to investigate other Co-based materials for LEOR applications, which could improve their stability while keeping the high selectivity or increasing it, if possible.

## Supplementary Material



## Notes

Lignin size characterization by gel permeation chromatography (GPC) and electrochemical surface area (ECSA) investigation for all Co electrodeposition procedures and for the selected catalysts after lignin oxidation (PDF)

## Abbreviations

LEOR: lignin electrochemical oxidation reaction
H: *p*-coumaryl alcohol
G: coniferyl alcohol
S: sinapyl alcohol
FE: Faradaic efficiency
XRD: X-ray diffraction
PDFs: powder diffraction file patterns
SEM: scanning electron microscopy
OER: oxygen evolution reaction
GPC: gel permeation chromatography
ECSA: electrochemical surface area
GC-FID: gas chromatography—flame ionization detector
