# Supplementary material for: Electrocatalytic Oxidation of Lignin on Cobalt Electrodes in Alkaline Media
Source: Langmuir. 2026 Jun 16;42(31):22550–60. doi: 10.1021/acs.langmuir.6c01244 (PMC13472163; doi:10.1021/acs.langmuir.6c01244)
Supplement: Supplementary file 1 [file la6c01244_si_001.pdf]

Supporting Information

Electrocatalytic Oxidation of Lignin on Cobalt Electrodes in Alkaline Media

André H. B. Dourado[1,2,], Lucas D. Germano[2], Lívia C. S. Epifanio[1], Matheus Santos[1], Antonio A. S. Curvelo[1], Hamilton Varela[1,*]*

[1] Instituto de Química de São Carlos, Universidade de São Paulo, 13566-590, São Carlos, SP, Brazil.

[2]Instituo de Química, Universidade Estadual Paulista "Júlio de Mesquita Filho", 14800-060, Araraquara, SP, Brazil.

* Corresponding authors: Prof. Dr. André H. B. Dourado (andre.dourado@unesp.br), and Prof. Dr. Hamilton Varela (hamiltonvarela@usp.br).

Table of contents

Figure S1: GPC chromatogram……………………………………………………...S3

Figure S2: i/E profiles at different scan rates of fresh film electrodeposited at -0.7 V…S4

Figure S3: i/E profiles at different scan rates of fresh film electrodeposited at -0.8 V..…S4

Figure S4: i/E profiles at different scan rates of fresh film electrodeposited at -0.9 V......S5

Figure S5: j/ν profiles based on Figures S2-S4……………….............……………......S5

Figure S6: j/E profiles at low scan rate of film electrodeposited at -0.7 V in KOH........S6

Figure S7: j/E profiles at low scan rate of film electrodeposited at -0.8 V in KOH........S6

Figure S8: j/E profiles at low scan rate of film electrodeposited at -0.9 V in KOH........S7

Figure S9: j/E profiles of film at -0.8 V in presence and absence of lignin......................S8

Figure S10: i/E profiles at different scan rates after LEOR at 0.8 V_{RHE}..........................S8

Figure S11: i/E profiles at different scan rates after LEOR at 1.0 V_{RHE}..........................S9

Figure S12: i/E profiles at different scan rates after LEOR at 1.6 V_{RHE}..........................S9

Figure S13: Derivative Tafel slope obtained from Fig.2d...S10

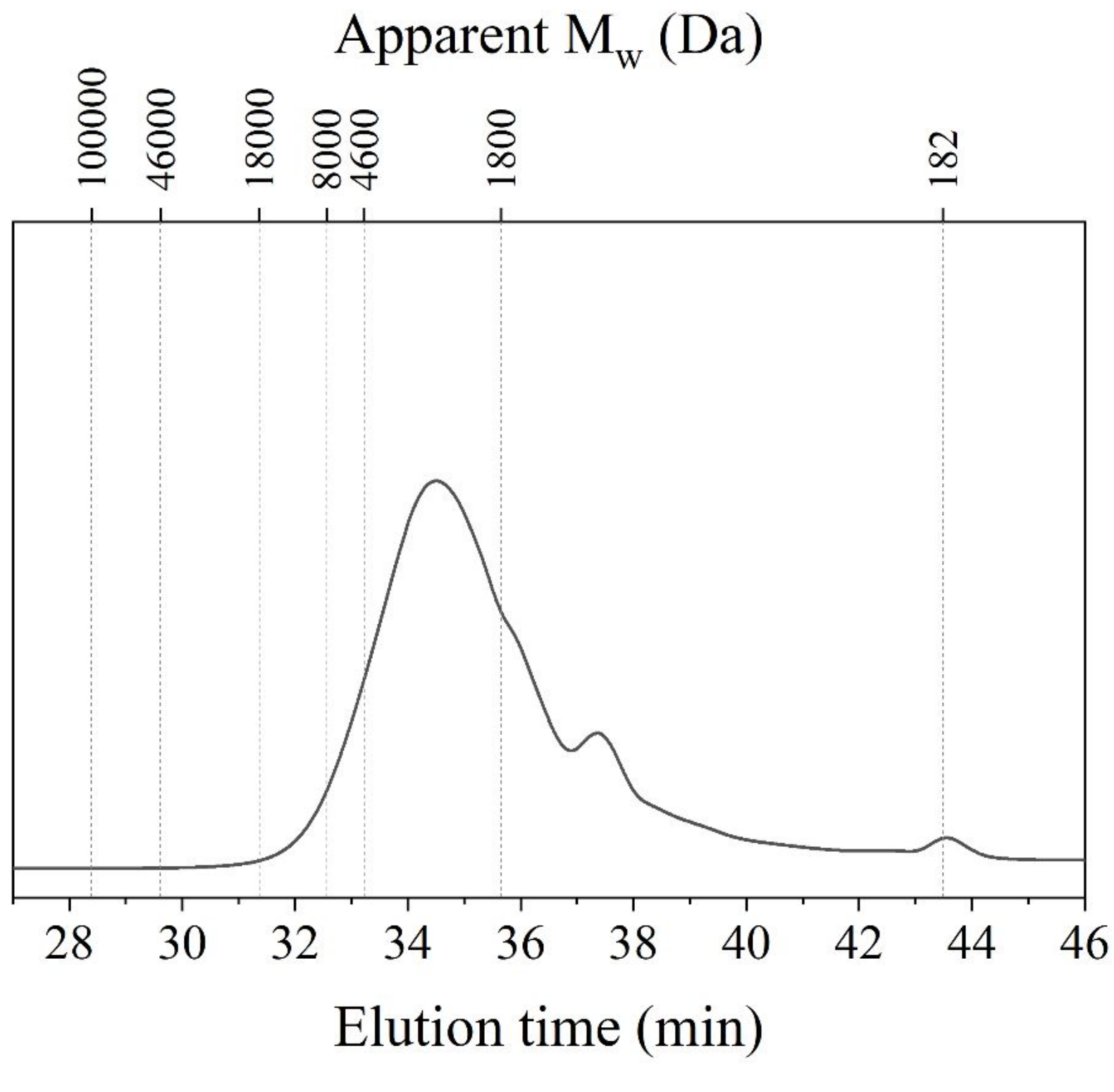

Figure S1. GPC chromatogram of industrial Kraft lignin isolated from Eucalyptus.

The chromatogram presents a large range of molecular weight distribution, from approximately 18000 Da to 182 Da, with average Mw, Mn and dispersity index of 2750 Da, 1300 Da and 2.1, respectively. Interestingly, chromatogram shows small peak at 44 minutes indicating the presence of monophenols species.

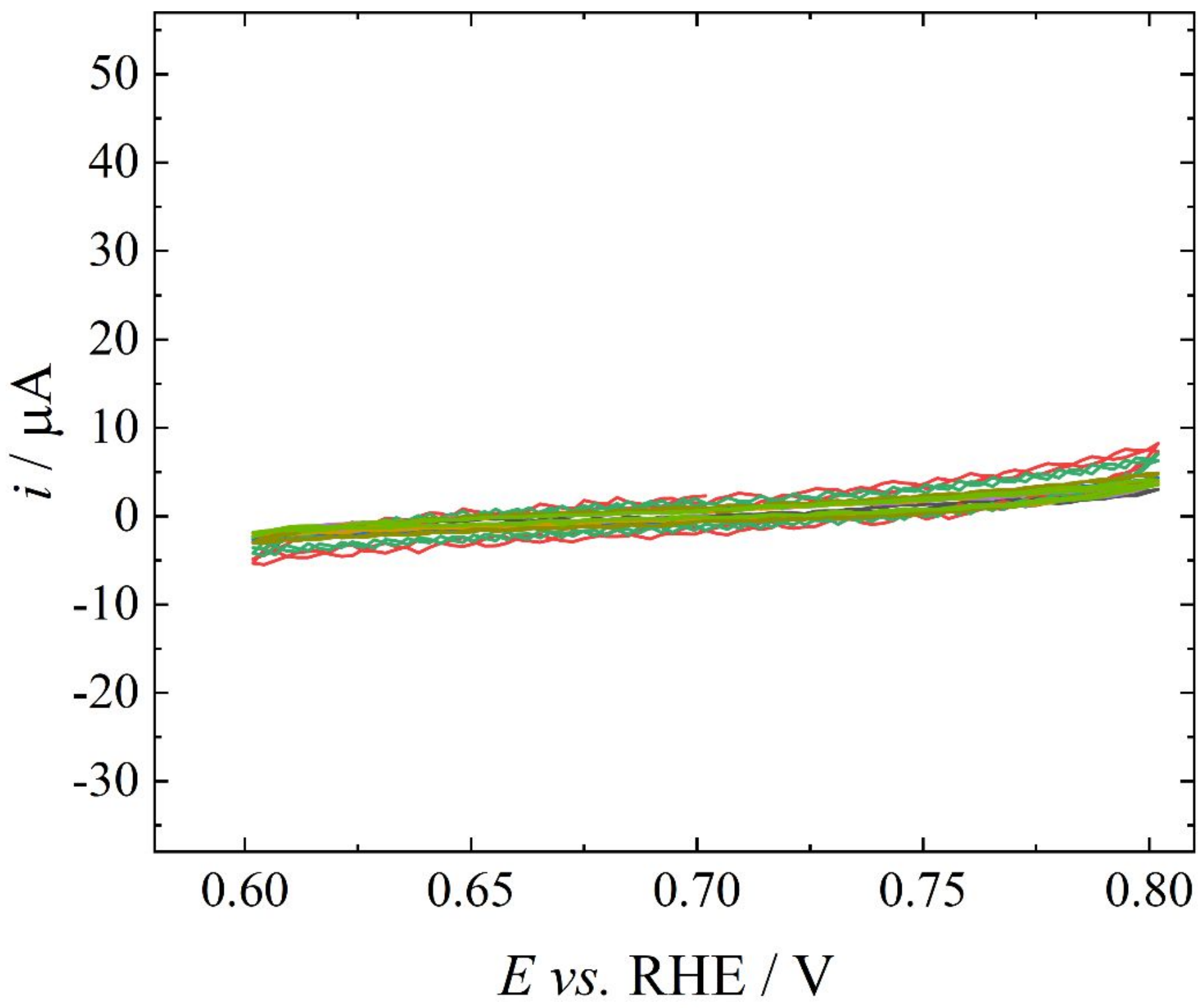

Figure S2. i/E profiles obtained at different scan rates (5 – 1000 mV s^{-1}) in KOH 2 mol L^{-1} for the freshly deposited Co electrode at -0.7 V *vs.* Ag/AgCl.

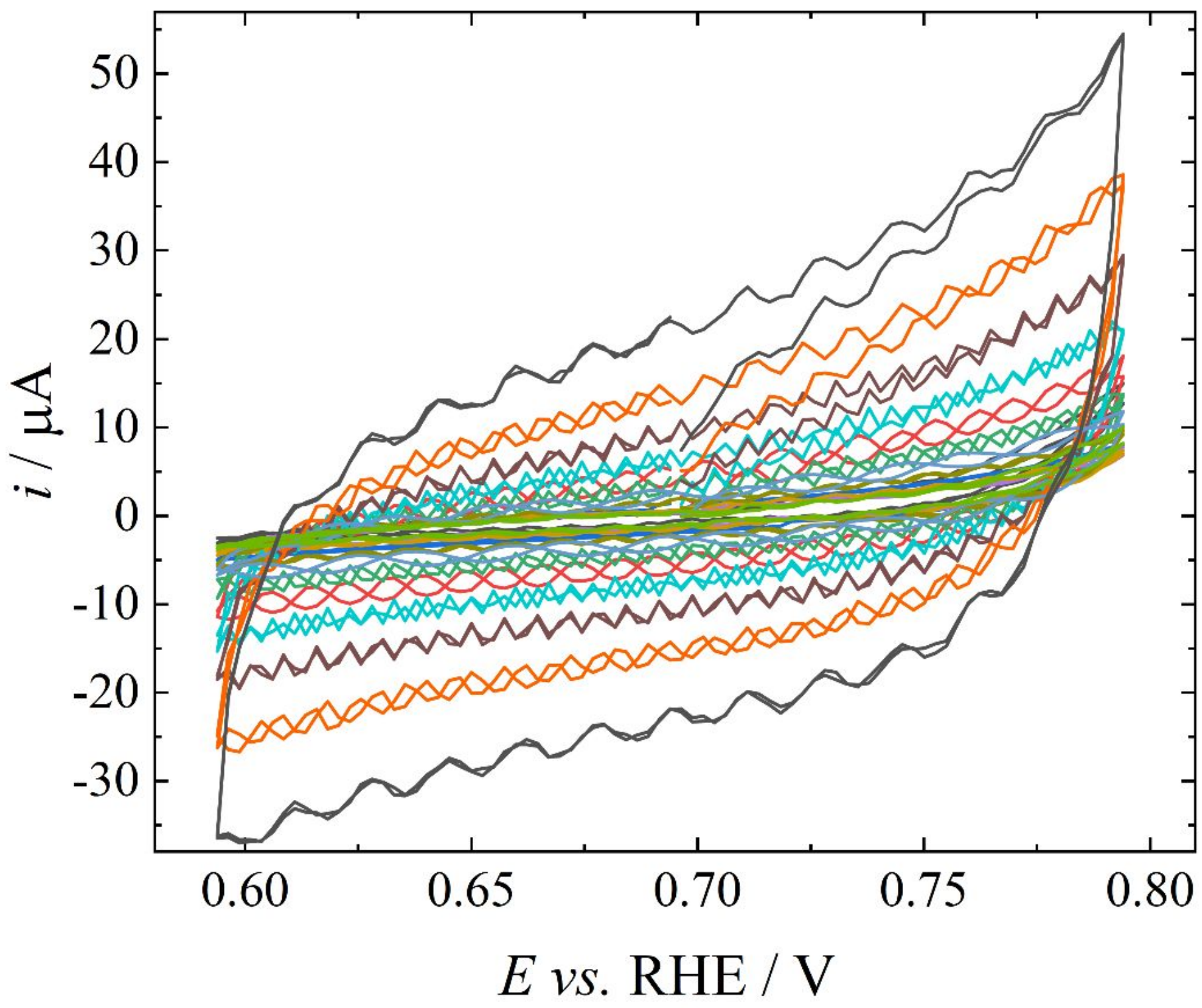

Figure S3. i/E profiles obtained at different scan rates (5 – 1000 mV s^{-1}) in KOH 2 mol L^{-1} for the freshly deposited Co electrode at -0.8 V *vs.* Ag/AgCl.

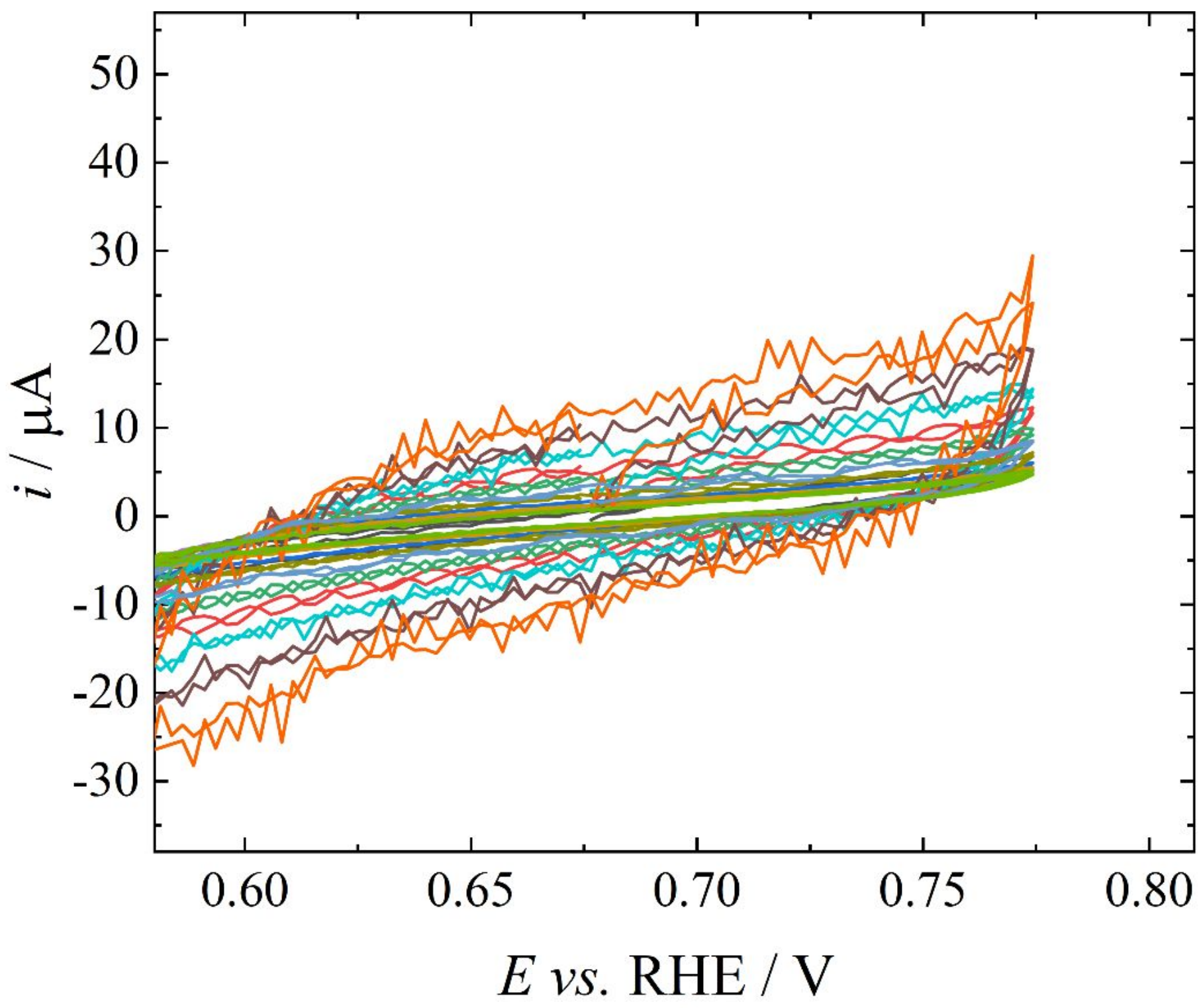

Figure S4. i/E profiles obtained at different scan rates (5 – 1000 mV s^{-1}) in KOH 2 mol L^{-1} for the freshly deposited Co electrode at -0.9 V *vs.* Ag/AgCl.

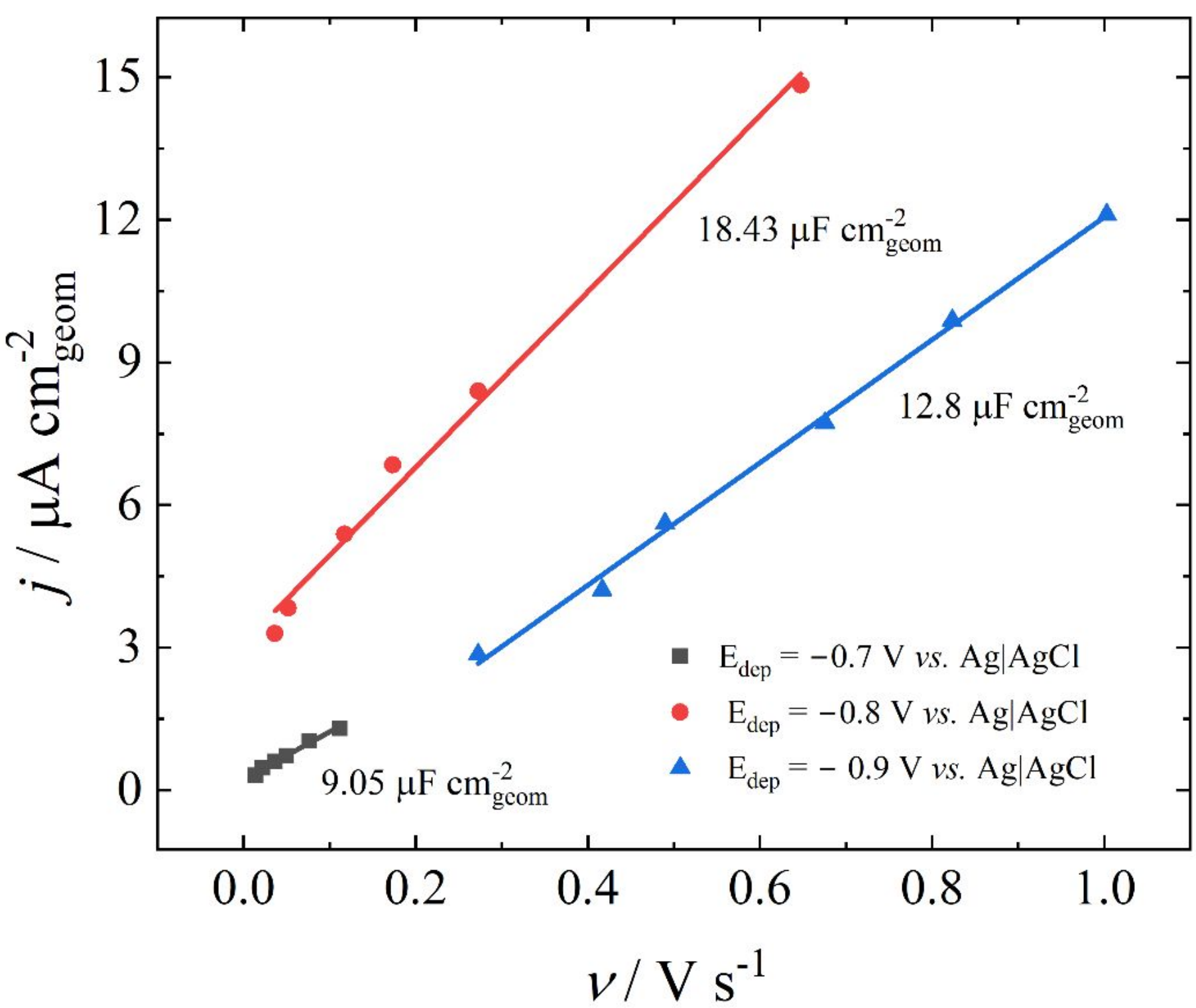

Figure S5. j/ν profiles obtained based on Figures S2-S4, showing the measured capacitances for the three electrodes. All data was normalized by the electrode geometrical area, 0.52 cm^{-2}.

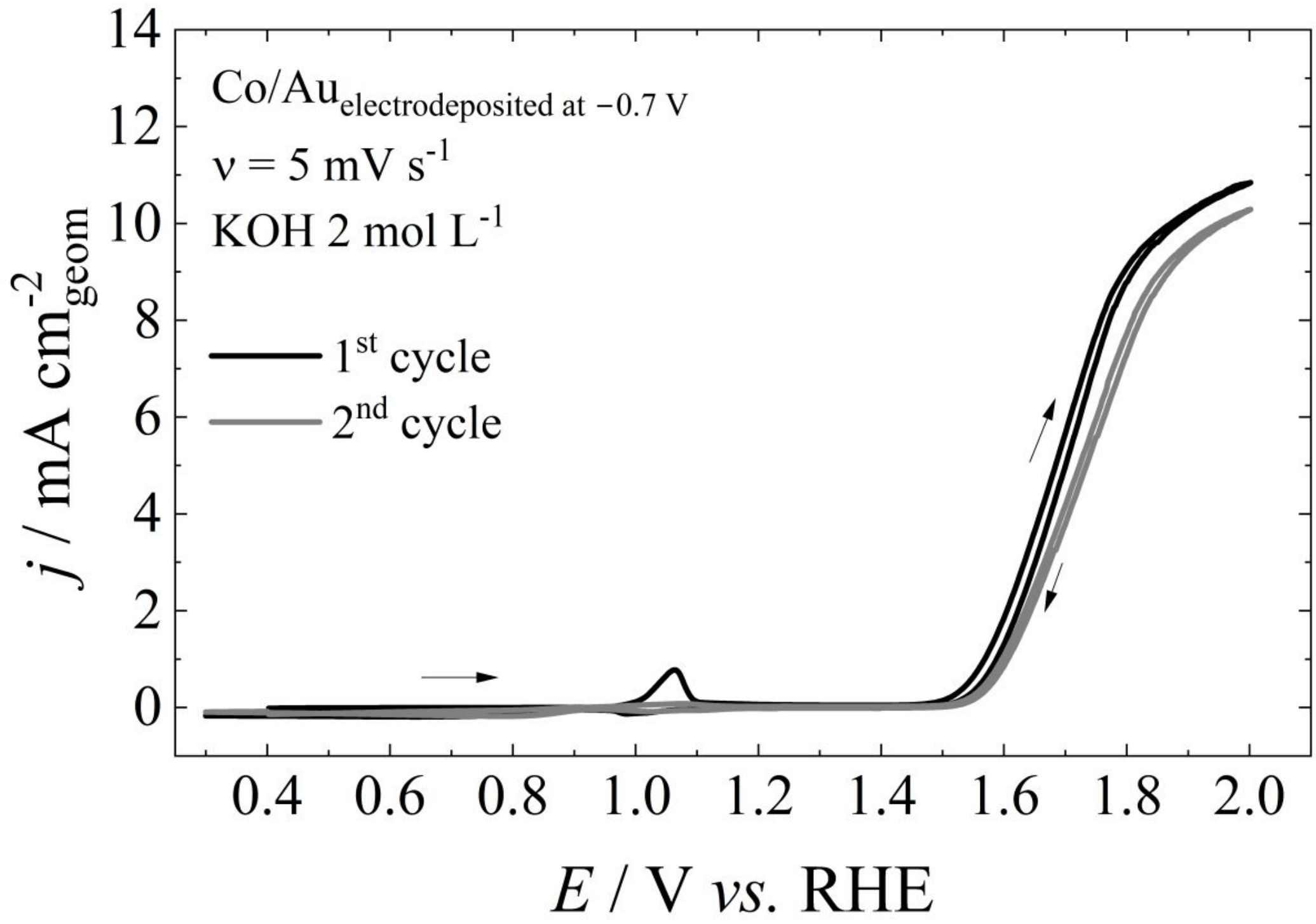

Figure S6. j/E profiles obtained at 5 mV s^{-1} in 2 mol L^{-1} KOH for the freshly deposited Co electrode at − 0.7 V *vs.* Ag/AgCl.

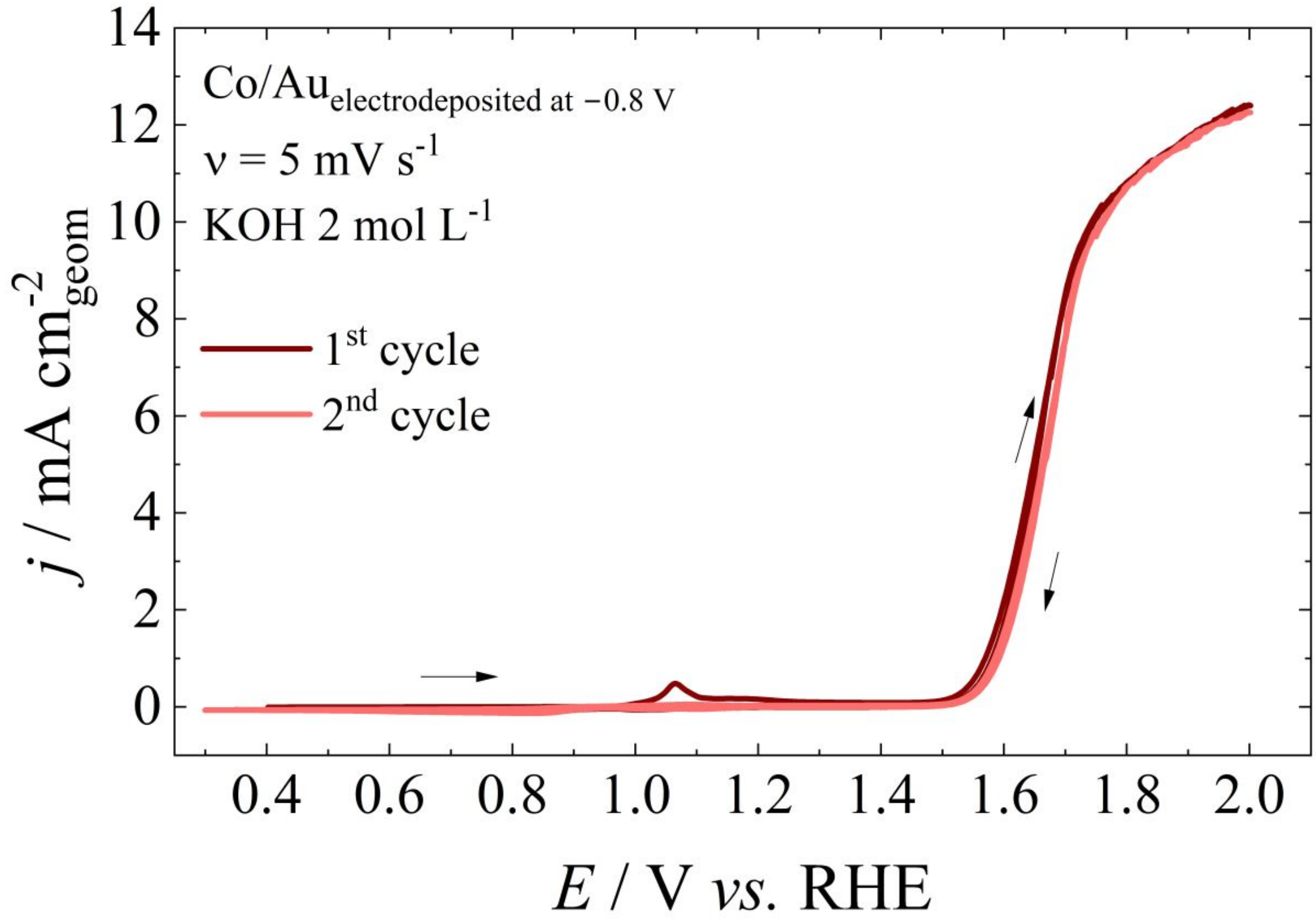

Figure S7 j/E profiles obtained at 5 mV s^{-1} in 2 mol L^{-1} KOH for the freshly deposited Co electrode at − 0.8 V *vs.* Ag/AgCl.

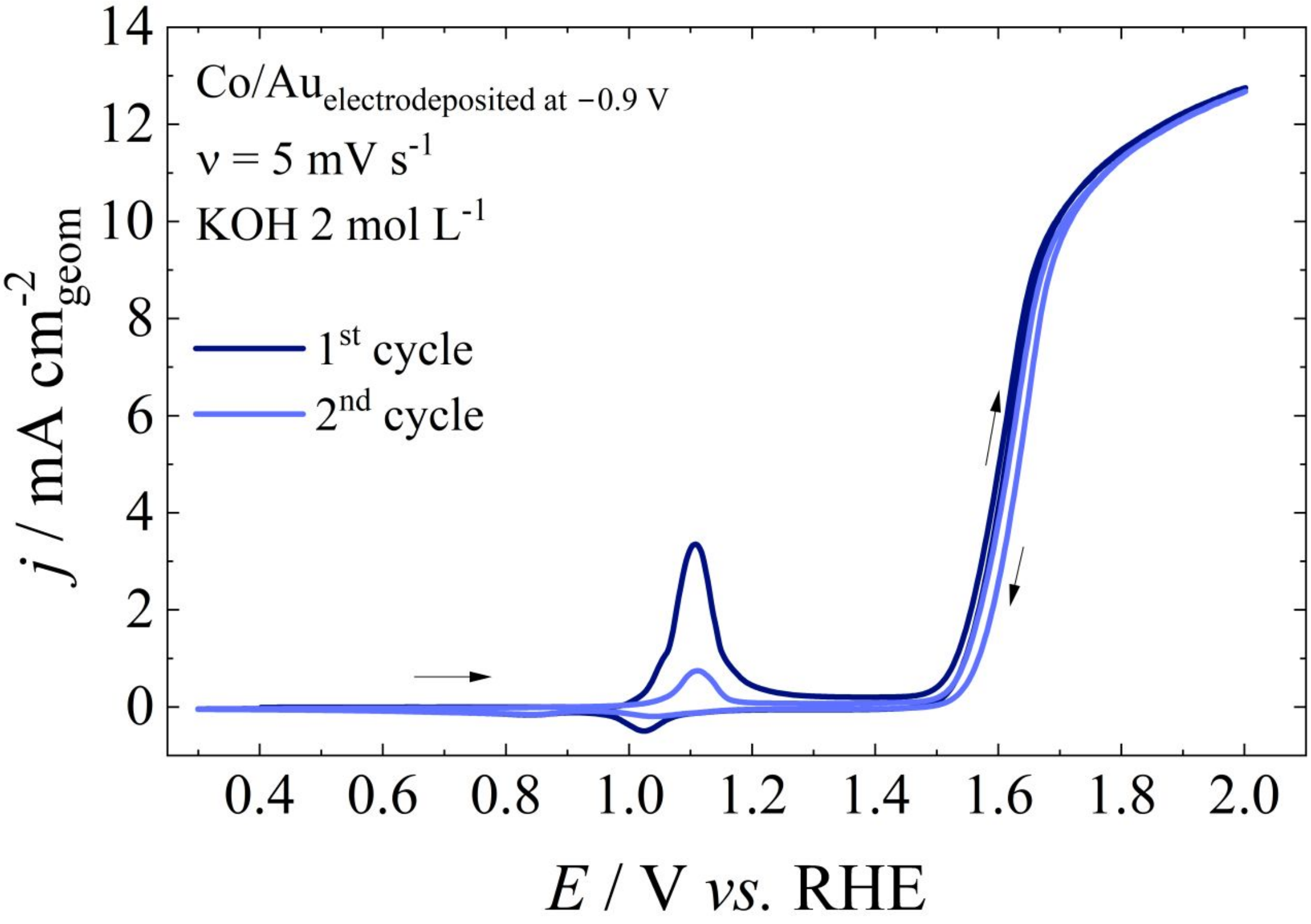

Figure S8. j/E profiles obtained at 5 mV s^{-1} in 2 mol L^{-1} KOH for the freshly deposited Co electrode at − 0.9 V *vs.* Ag/AgCl.

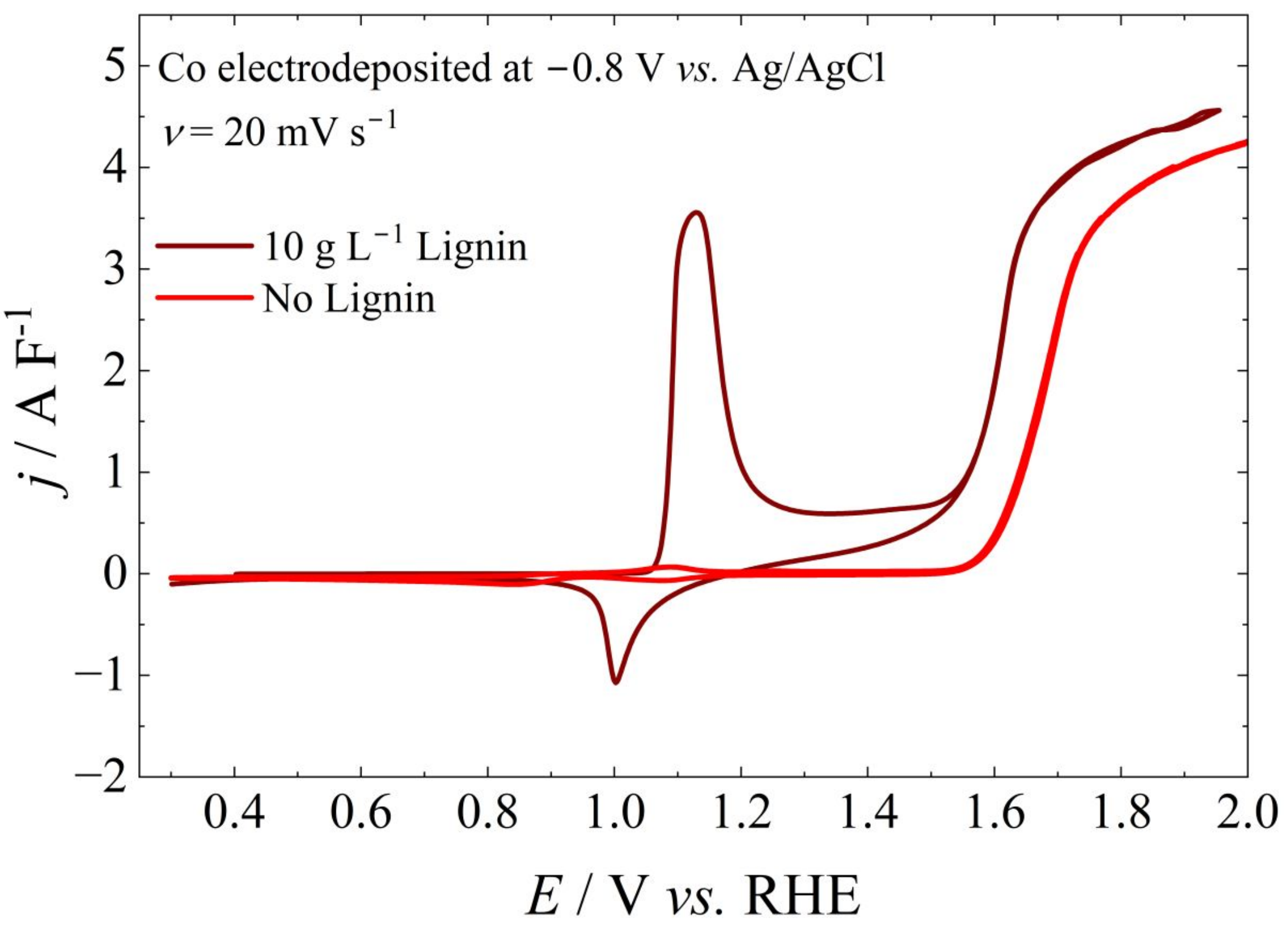

Figure S9: j/E profiles of freshly prepared Co/Au (E_{ed} = − 0.8 $V_{Ag/AgCl}$) at ν = 20 mV s^{-1} in 2 mol L^{-1} KOH, with (brown solid line) and without (red solid line) lignin. Ar purged during all the experiments.

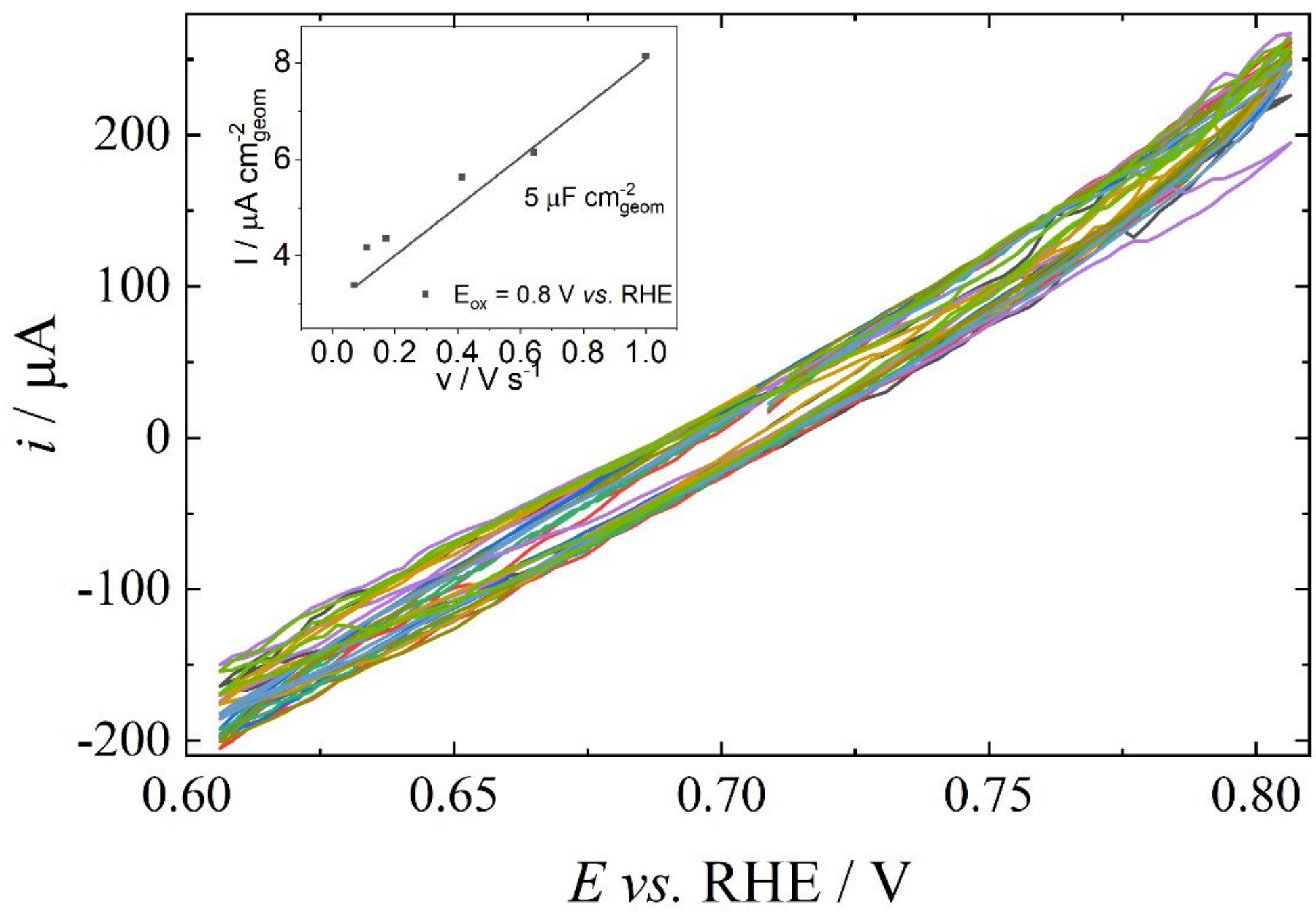

Figure S10. i/E profiles obtained at different scan rates (5 – 1000 mV s^{-1}) in KOH 2 mol L^{-1} for the Co electrode after LEOR at 0.8 V_{RHE}. Insert: j/ν profile and the corresponding surface capacitance, corrected by the electrode geometrical area.

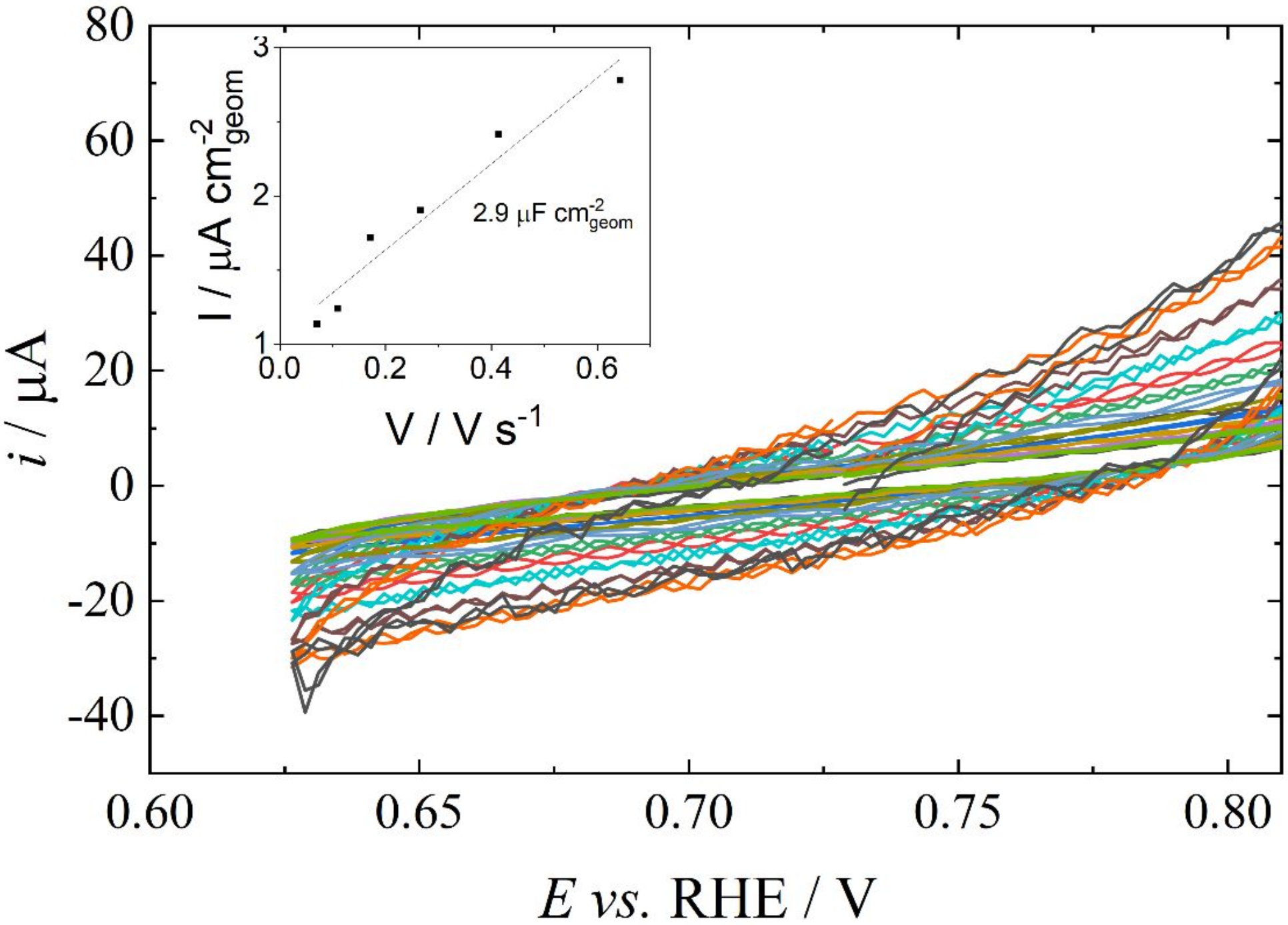

Figure S11. i/E profiles obtained at different scan rates (5 – 1000 mV s^{-1}) in KOH 2 mol L^{-1} for the Co electrode after LEOR at 1.0 V_{RHE}. Insert: j/ν profile and the corresponding surface capacitance, corrected by the electrode geometrical area.

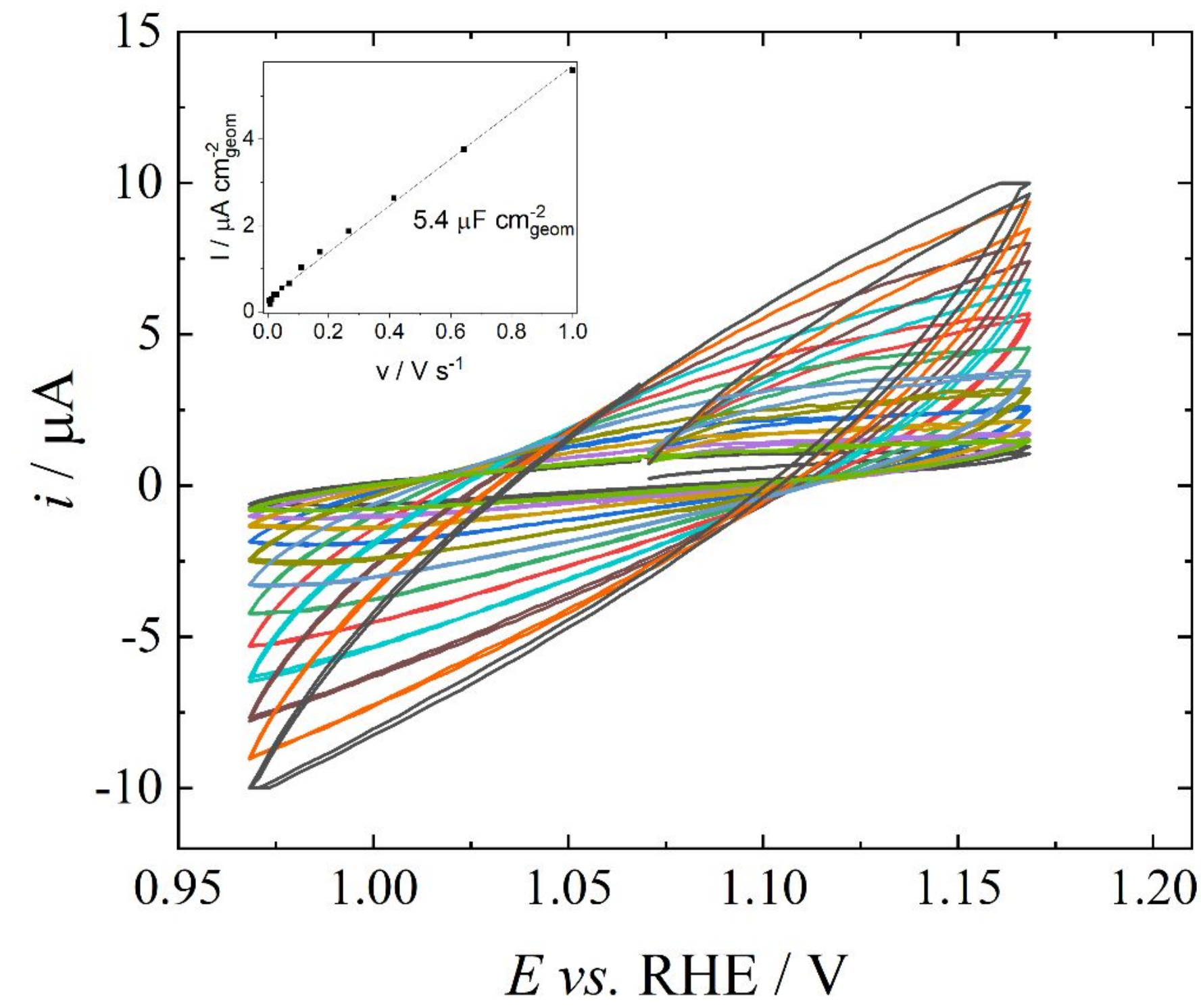

Figure S12. i/E profiles obtained at different scan rates (5 – 1000 mV s^{-1}) in KOH 2 mol L^{-1} for the Co electrode after LEOR at 1.6 V_{RHE}. Insert: j/ν profile and the corresponding surface capacitance, corrected by the electrode geometrical area.

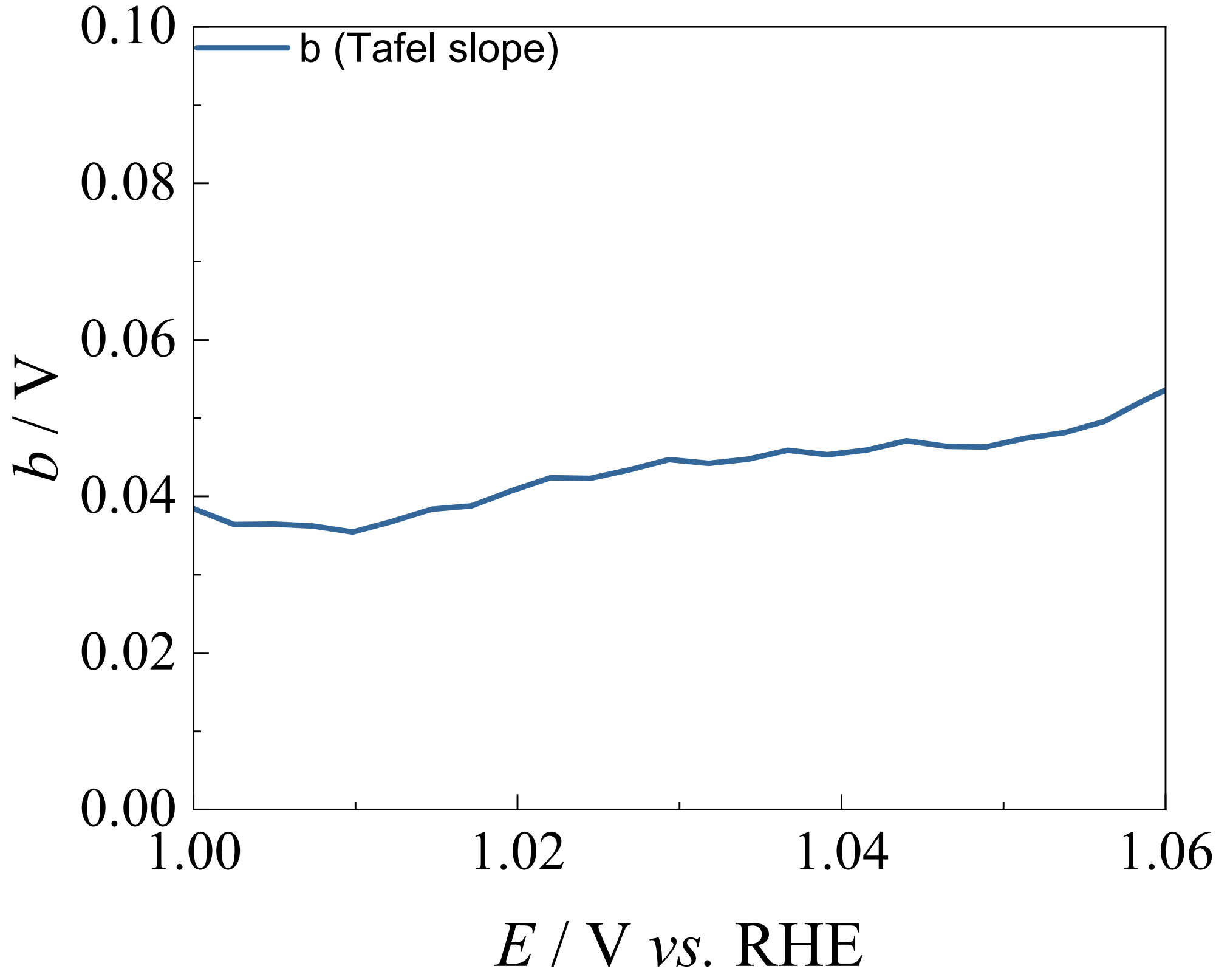

Figure S13. Derivative Tafel slope obtained for the Co electrode deposited at -0.80 $V_{Ag/AgCl}$, based on the polarization curve shown at Figure 2d.
